# Innate and adaptive type 2 immune cell responses in genetically controlled resistance to intestinal helminth infection

**DOI:** 10.1038/icb.2013.109

**Published:** 2014-02-04

**Authors:** Kara J Filbey, John R Grainger, Katherine A Smith, Louis Boon, Nico van Rooijen, Yvonne Harcus, Stephen Jenkins, James P Hewitson, Rick M Maizels

**Affiliations:** 1 Institute for Immunology and Infection Research, University of Edinburgh, Edinburgh, UK; 2 Bioceros BV, Utrecht, The Netherlands; 3 Department of Molecular Cell Biology and Immunology, VU Medical Center, Amsterdam, The Netherlands

**Keywords:** macrophage, nematode, Th2 cytokine

## Abstract

The nematode *Heligmosomoides polygyrus* is an excellent model for intestinal helminth parasitism. Infection in mice persists for varying lengths of time in different inbred strains, with CBA and C57BL/6 mice being fully susceptible, BALB/c partially so and SJL able to expel worms within 2–3 weeks of infection. We find that resistance correlates not only with the adaptive Th2 response, including IL-10 but with activation of innate lymphoid cell and macrophage populations. In addition, the titer and specificity range of the serum antibody response is maximal in resistant mice. In susceptible strains, Th2 responses were found to be counterbalanced by IFN-γ-producing CD4^+^ and CD8^+^ cells, but these are not solely responsible for susceptibility as mice deficient in either CD8^+^ T cells or IFN-γ remain unable to expel the parasites. Foxp3^+^ Treg numbers were comparable in all strains, but in the most resistant SJL strain, this population does not upregulate CD103 in infection, and in the lamina propria the frequency of Foxp3^+^CD103^+^ T cells is significantly lower than in susceptible mice. The more resistant SJL and BALB/c mice develop macrophage-rich IL-4Rα-dependent Type 2 granulomas around intestinal sites of larval invasion, and expression of alternative activation markers Arginase-1, Ch3L3 (Ym1) and RELM-α within the intestine and the peritoneal lavage was also strongly correlated with helminth elimination in these strains. Clodronate depletion of phagocytic cells compromises resistance of BALB/c mice and slows expulsion in the SJL strain. Thus, Type 2 immunity involves IL-4Rα-dependent innate cells including but not limited to a phagocyte population, the latter likely involving the action of specific antibodies.

Immunity in the gut has evolved to minimize immune reactivity to commensal bacteria and food antigens, while remaining alert to incoming pathogenic organisms.^1, 2, 3^ Many helminth parasites are adept at entering this environment, as is evident from the fact that >2 billion people are currently infected with intestinal hookworm, whipworm and ascarid nematodes.^4^ A major challenge for global health, therefore, is to understand how parasites engage with the finely balanced homeostatic system of the gastrointestinal tract in order to establish themselves for long-term infection.

Studies in both mouse and human populations strongly indicate that immunity to gastrointestinal nematode parasites requires a strong Th2 responsiveness profile^5, 6, 7, 8^ with the canonical type 2 cytokines IL-4 and IL-13 critical in mobilizing a raft of innate effector mechanisms that disable and expel gut helminths.^6, 8, 9, 10, 11, 12^ Interestingly, human populations show a spectrum of responses to helminth infection, varying from effective resistance through hyporesponsiveness and tolerance to hyperreactivity and pathogenesis.^13^ Allelic variation at key loci controlling type 2 cytokines and their signals is an important genetic factor influencing the outcome of helminth infection,^14, 15^ and likewise different strains of mice can display contrasting patterns of susceptibility or resistance to helminth parasites. Hence, mouse models can provide new insights not only into immunological mechanisms of protection but also the genetic basis for variation in the efficacy of those mechanisms.

In one well-used model, the rat parasite *Nippostrongylus brasiliensis* is rapidly expelled by Th2-dependent mechanisms in all immunocompetent strains of mice.^16^ A more balanced picture is seen with the cecal-dwelling *Trichuris muris*, in which Th2-dependent immunity is directly antagonized by the degree of Th1 responsiveness.^10^ Thus, blocking IFN-γ responses enables a susceptible mouse to clear infection,^9^ whereas exogenous IL-12 prolongs infection in a genotypically resistant animal.^17^ A further layer of complexity is observed with the duodenal parasite *Heligmosomoides polygyrus*, which generates a significant regulatory T-cell population that inhibits Th2 immunity.^18, 19, 20, 21, 22^ Recent data implicate similar regulatory effects in human intestinal helminth infections,^23, 24, 25, 26^ suggesting that *H. polygyrus* may offer a valuable system to model such interactions.

*H*. *polygyrus* is a natural mouse parasite that is able to establish primary infections in most laboratory mouse strains of mice.^27, 28^ Drug-mediated worm clearance of susceptible mice, however, results in protective immunity against secondary infection, acting against the larval stage, which enters the gut wall for ∼8–10 days before emerging into the lumen as mature adults.^27^ Resistance to reinfection operates through an IL-4R-dependent population of alternatively activated macrophages that populate granulomatous cysts around larvae in the intestinal wall.^12, 29^ In addition, secondary immunity requires IgG1 antibody responses, and a level of protection can be conferred by passive transfer of this isotype.^30, 31, 32, 33, 34^

A further model of immunity in *H. polygyrus,* exploits the fact that inbred strains differ markedly in their ability to expel primary infections.^28, 35, 36, 37, 38^ Resistant mouse strains such as SJL show faster and stronger antibody and Th2-type responses,^39, 40^ but as yet few details are available that compare T cell subsets or innate immune components between strains with differing capacity to reject primary infection. In addition, while resistance in previously immunized mice is associated with the formation of granulomas around encysted larvae,^12, 29^ the role of granulomas in primary immunity has not been evaluated. We accordingly set out to compare the immunological phenotypes in four well-characterized strains of mice that offer a spectrum of susceptibility to *H. polygyrus*, demonstrating that intensity of both innate and adaptive type 2 cell responses correlate with the resistant state, while susceptibility is associated with increased Treg activation, IFN-γ expression by CD4^+^ and CD8^+^ T cells, as well as the production of IgE.

## Results

### Graded resistance associated with early reduced fecundity and granuloma development

**Figure 1:**
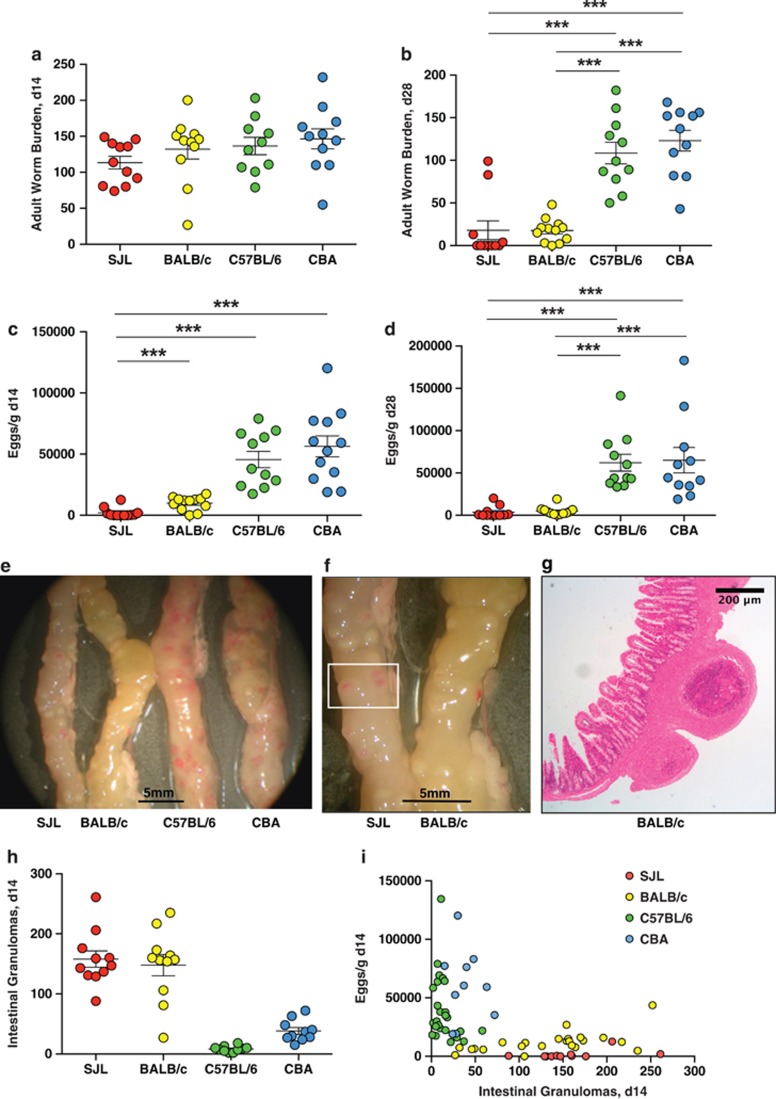
Variation in susceptibility to primary infection with *H. polygyrus* manifests first with early differences in parasite fecundity, and subsequent loss of adult worms. Age-matched female SJL, BALB/c, C57BL/6 and CBA mice were infected with 200 *H. polygyrus* L3 larvae by gavage. Data presented are pooled from two independent experiments. Bars in **a**–**d** and **h** indicate means and standard errors of the mean. (**a**, **b**) Luminal adult parasites at day 14 and 28 of infection. (**c**, **d**) Fecal egg counts at day 14 and 28 of infection. (**e**, **f**) Representative images of d14 intestinal granulomas in different mouse strains. Scale bars show 5 mm. (**g**) Representative image of intestinal granuloma in a d14-infected BALB/c mouse, hemotoxylin and eosin-stained. Scale bar shows 200 μm. (**h**) Number of granulomas in small intestine in different strains of mice at day 14 of infection. (**i**) Negative relationship between egg numbers and granulomas at day 14 of infection. Statistically significant differences are indicated; ***P<0.001. A full colour version of this figure is available at the *Immunology and Cell Biology* journal online.

In primary infection with the gastrointestinal nematode *H. polygyrus*, larvae first invade the duodenal wall and emerge 8–10 days later as adult worms into the lumen of the gut. Inbred mouse strains are known to vary significantly in their susceptibility to this parasite,^28, 35, 36, 37, 38^ and in comparing four strains we noted three critical features of differential immunity. First, luminal adult worms were present in all groups at day 14, soon after emergence (Figure 1a), and only subsequently did genetically determined differences in expulsion become apparent. Thus, by day 28 resistant strains had expelled most of the adult parasites, with BALB/c and SJL mice carrying the lowest numbers, whereas C57BL/6 and CBA retained significantly higher loads (Figure 1b).

Secondly, at the earlier time point the stronger immune phenotype was clearly manifest in terms of egg production, which at day 14 was much lower in relatively resistant SJL mice than in the fully susceptible CBA and C57BL/6 strains (Figure 1c). As the total number of adult worms in the gut lumen was similar in all groups at this time point (Figure 1a), this phase of immunity represents a reduction in worm fitness as reflected by their fecundity. By day 28, egg production more closely mirrored adult worm loads with CBA and C57BL/6 mice excreting the most, and BALB/c and SJL the fewest (Figure 1d).

Thirdly, the more resistant strains showed more extensive development of macroscopic granulomas in the intestinal wall (Figures 1e–g), which although numerous in the more resistant genotypes are sparse in the fully susceptible mice (Figure 1h). The abundance of granulomas shows, in this comparison, an inverse relationship with worm fecundity (Figure 1i), suggesting that they may impair fitness of the parasite while in the intestinal wall.

### Resistance follows a gradient of higher Type 2 responses and lower Type 1

**Figure 2:**
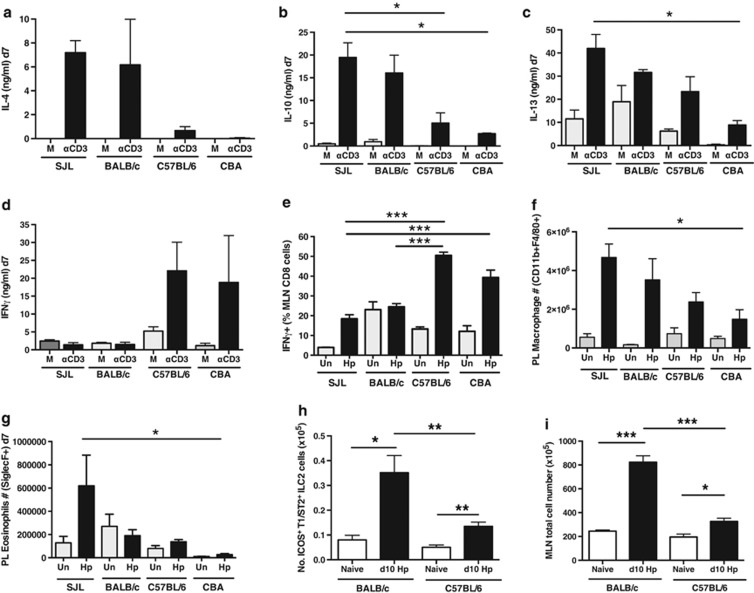
Profile of MLN and peritoneal cells during *H. polygyrus* infection of different mouse strains. MLNC from naive (Un) or day 7 *H. polygyrus*-infected female SJL, BALB/c, C57BL/6 and CBA were stimulated *in vitro* with medium alone (M) or anti-CD3 antibody and supernatants assayed for secreted cytokines (**a**–**d**), or stained directly for intracellular IFN-γ (**e**) after 4 h of stimulation with PMA, ionomycin and brefeldin. Also shown are the number of CD11b^+^F4/0^+^ macrophages and SiglecF^+^ eosinophils stained in the peritoneal lavage of day 7infected mice (**f**, **g**) and lineage-negative innate lymphoid cells from the MLN-expressing intracellular GATA-3 and surface CD127, ICOS and T1/ST2 (**h**) from day 10-infected mice, with total MLN cell number also shown (**i**). Data in **a**-**e** represent mean values±standard errors, from ⩾3 individual mice of each strain, from one of 4 independent experiments with similar results; data in **f**–**i** are pooled from two independent experiments with 3–6 mice in each group and show means with standard errors. Further data on T cell cytokine profiles are presented in Supplementary Figures 1 and 2 and on the gating of innate lymphoid cells in Supplementary Figure 3. (**a**–**d**) Polyclonal cytokine release by anti-CD3 stimulated MLNC—IL-4, IL-10, IL-13 and IFN-γ. (**e**)Intracellular staining for IFN-γ among CD8^+^ MLN T cells. (**f**) Macrophage (CD11b^+^F4/80^+^) numbers in the peritoneal lavage of naive and d7-infected mice. (**g**) SiglecF^+^ cell numbers in the same peritoneal lavage samples. (**h**) Number of ICOS^+^, T1/ST2^+^, CD127^+^, GATA-3^+^ lineage^-^ MLNC from naive and d10-infected mice. (**i**) Total MLN cell numbers from the same animals. Statistically significant differences are indicated; *P<0.05; **P<0.01; ***P<0.001.

Cellular immune responses were first assessed at day 7 post infection using both polyclonal and antigen-specific assays. *In vitro* stimulation of draining mesenteric lymph node (MLN) cells with anti-CD3 antibody elicited high levels of IL-4, IL-10 and IL-13 from SJL and BALB/c MLN cells (MLNC; Figures 2a–c). In the same assays, susceptible C57BL/6 and CBA mice mounted weaker Th2 responses, while expressing higher levels of IFN-γ (Figure 2d). A similar picture emerged from intracellular staining of CD4^+^ T cells for IL-4 (data not shown) and other type 2 cytokines (Supplementary Figures 1A–C).

Antigen-specific T-cell responses in all four strains of mice were evaluated by *in vitro* stimulation with *H. polygyrus* excretory-secretory antigen (HES). At day 7 post infection, all strains mounted type 2 cytokine responses that were sustained past day 14 only in the more resistant strains and suppressed in the susceptible strains (Supplementary Figures 2A–C). The association with resistance was more evident for IL-4 and IL-10 than for IL-13. In contrast, Th-17 responses were variable and did not associate with resistance phenotype (Supplementary Figure 2D) and only susceptible strains generated early and sustained IFN-γ production in response to HES stimulation (Supplementary Figure 2E). IFN-γ responses were also present within a substantial proportion of CD8^+^ MLNC (Figure 2e; Supplementary Figure 1F) from the susceptible strains.

Notably, the strength of the IL-10 response, both at the polyclonal level (Figure 2b; Supplementary Figure 1B) and in terms of HES-specific cytokine release (Supplementary Figure 2B) was strongly associated with resistance, with maximal levels in the most resistant SJL strain at all time points assayed. However, no differences were seen in IL-9 responses following HES challenge *in vitro* (Supplementary Figure 2F), although over-expression of IL-9 has been reported to promote expulsion of *H. polygyrus*.^10^

We then measured the profile of key innate cell populations during infection. Within the peritoneal cavity, a major site of macrophage activation,^41^ CD11b^+^F4/80^+^ macrophages expanded dramatically in all strains, but significantly more so in the two resistant strains (Figure 2f). Only SJL mice recruited large eosinophil numbers to this site (Figure 2g). At day 7, variable numbers of IL-5 and 13-expressing type 2 innate lymphoid cells (ILC2s lacking CD3, CD4, CD8α, CD11c, CD19, DX5, F4/80, Gr1 and MHC class II) were observed in the SJL, which did not achieve statistical significance (data not shown). However, a more robust difference was noted by day 10 between the resistant BALB/c and susceptible C57BL/6 mice in the number of ILC2s, when defined by expression of ICOS, T1/ST2 and CD127, and staining for intracellular GATA3 as a marker of ILC2 commitment, as illustrated in Supplementary Figure 3.^42, 43^ Notably, while ILC2 numbers were significantly higher in the BALB/c strain (Figure 2h) in both genotypes these showed similar expansion to total cell numbers in the MLN (Figure 2i).

### Resistance requires IL-4R signaling, but susceptibility is not dependent on CD8^+^ T cells or IFN-γ

**Figure 3:**
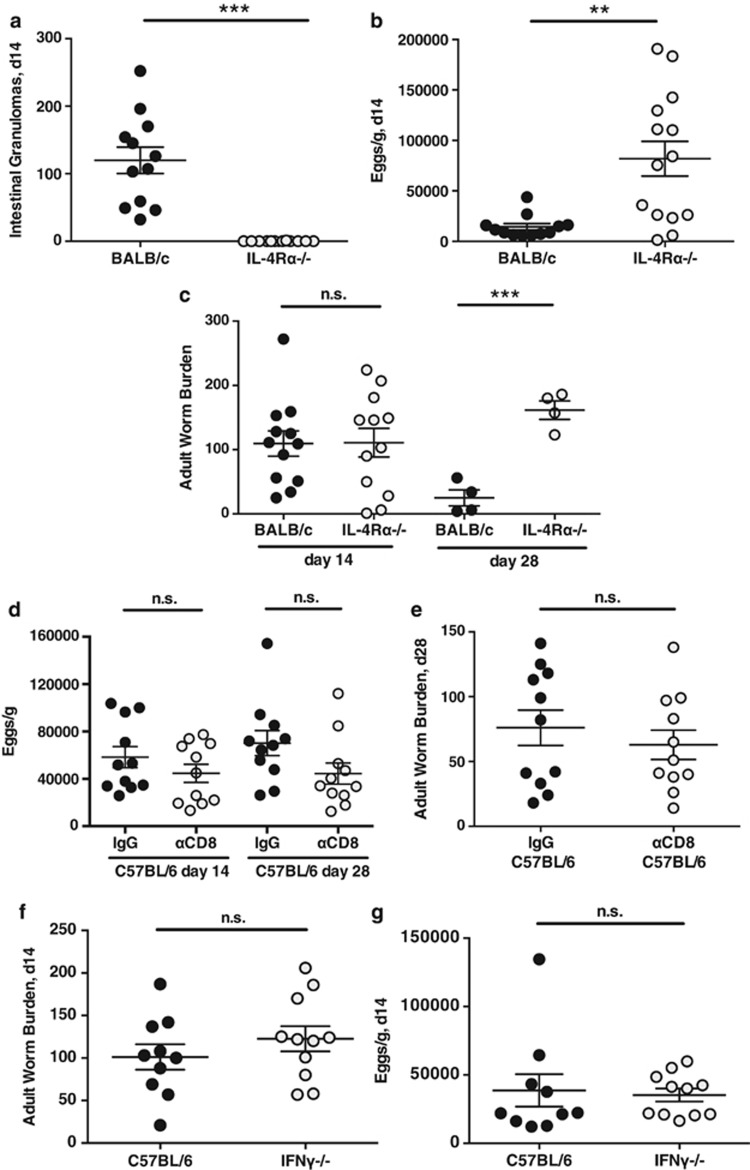
Immunity to *H. polygyrus* is completely dependent on IL-4Rα-mediated signaling but is not significantly influenced by CD8 T cells and IFN-γ. (**a**) Ablation of intestinal granuloma formation in IL-4Rα-deficient and BALB/c wild-type mice. Data in this and other panels show means and standard errors. (**b**, **c**) Egg production and adult worm loads in IL-4Rα-deficient and BALB/c wild-type mice. (**d**, **e**) Effect of anti-CD8 antibody depletion on egg production and adult worm load following infection in C57BL/6 mice. (**f**, **g**) Adult worm loads and egg production and in IFN-γ-deficient and C57BL/6 wild-type mice. Statistically significant differences are indicated; **P<0.01; ***P<0.001.

It is well established that resistance to gastrointestinal nematodes requires IL4R-mediated signaling in both hematopoietic and non-hematopoietic cells.^16, 44^ To test whether granuloma development was similarly dependent, BALB/c and congenic IL-4Rα-deficient mice were infected. Very clearly, no granulomas developed in the IL-4Rα^−/−^ animals (Figure 3a) and, as expected, IL-4Rα-deficient mice were highly susceptible to infection with increased fecundity per worm (Figure 3b); they also failed to expel adult parasites by day 28 (Figure 3c).

In the susceptible backgrounds, we questioned whether high levels of IFN-γ expression within the CD4^+^ and CD8^+^ T cell subsets were responsible for their failure to expel *H. polygyrus*, as depletion of this cytokine from *Trichuris muris*-infected mice is sufficient to confer resistance in normally susceptible animals.^9^ First, we depleted CD8^+^ T cells from C57BL/6 mice with monoclonal antibody YTS169; as shown in Figure 3d, this resulted in modest reductions in egg counts, which did not attain statistical significance, while adult worm numbers were unchanged at day 28 (Figure 3e). Secondly, we infected IFN-γ-deficient mice on the C57BL/6 background: this genotype shows reduced worm loads at 28 days post infection^45^ but at the earlier time point of 14 days, no difference was observed in adult worm numbers (Figure 3f). Unlike earlier experiments,^45^ egg production at day 14 was also unaltered (Figure 3g), indicating that the effect of IFN-γ deficiency is not profound in the C57BL/6 setting. Hence, IFN-γ alone contributes to susceptibility but in contrast to the *T. muris* system, does not fully account for the failure of resistance in the susceptible mice.

### Activation of Foxp3^+^ Treg is subdued in resistant mice

*H. polygyrus* has previously been reported to expand the numbers of Foxp3^+^ regulatory T cells in C57BL/6 and BALB/c mice,^19, 21^ and in particular to stimulate a significant rise in CD103 expression within the Foxp3^+^ T reg compartment.^19^ The induction of CD103 is considered an activation marker for Tregs^46^ and is strongly TGF-β-dependent in *H. polygyrus*-infected mice.^45^ Furthermore, interfering with TGF-β signaling in chronically infected mice has been shown to increase worm expulsion,^22^ although depletion of Tregs in Foxp3–diphtheria toxin receptor mice did not alter the worm burden at day 14.^47^

**Figure 4:**
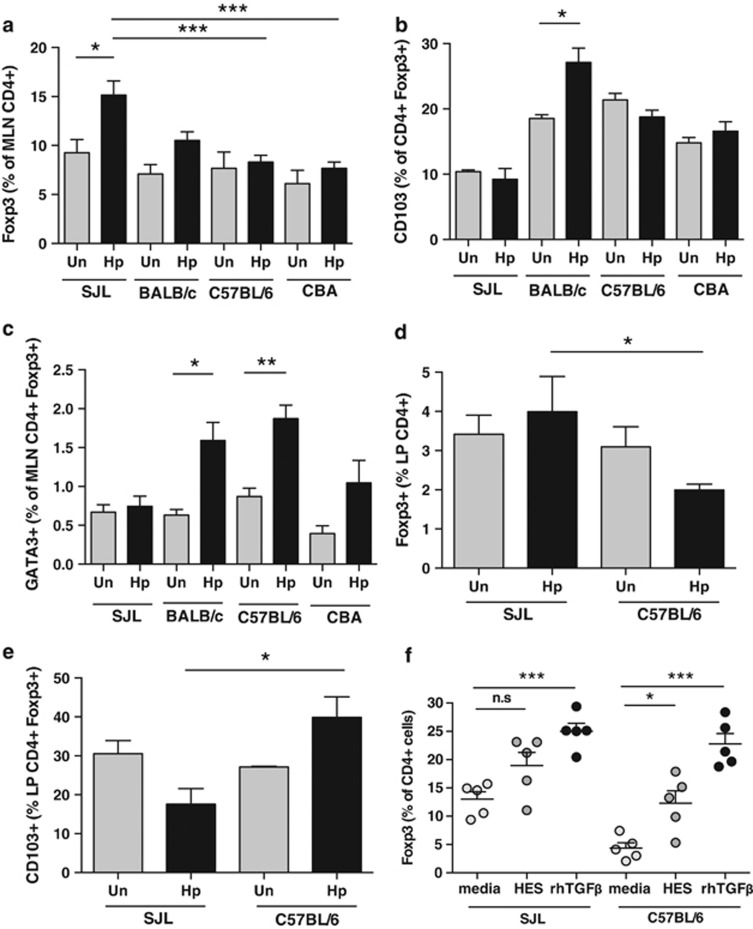
Qualitative differences in CD4^+^FoxP3^+^ regulatory T cells from SJL mice. CD4^+^ T cells from naive or day 7 *H. polygyrus*-infected female SJL, BALB/c, C57BL/6 and CBA mice were stained for expression of Foxp3 together with Helios, CD103 and GATA3. Results are pooled from two experiments. Positive cells are expressed as the mean with standard error from 2–6 animals per group. Experiments in **d** and **e** show LP cells extracted from SJL and C57BL/6 mice before and after a 7-day infection, and are pooled results from two experiments. (**a**) Foxp3 expression as a percentage of CD4^+^ cells in MLN of naive and d7 infected mice. (**b**) CD103 expression as a percentage of CD4^+^Foxp3^+^ cells in the MLN of naïve and d7 infected mice. (**c**) GATA3 staining of CD4^+^Foxp3^+^ cells in the MLN of naïve or d7 infected mice. (**d**) Foxp3 expression of CD4^+^ LP cells. (**e**) CD103 expression as a percentage of CD4^+^Foxp3^+^ cells in the LP of naive and d7 infected mice. (**f**) Foxp3 expression as a percentage of CD4^+^ cells from splenocyte cultures for 72 h with media, 10 μg/ml HES or 10 ng/ml rhTGF-β. Statistically significant differences are indicated; *P<0.05; **P<0.01; ***P<0.001.

We therefore examined Foxp3^+^ T cell populations in different mice: perhaps surprisingly, the strain that exhibited the clearest increment in the percentage of MLN Foxp3^+^CD4^+^ Tregs as measured by flow cytometry was the most resistant SJL (Figure 4a). However, when this subset was further analyzed for the expression of CD103, it was notable that the SJL mouse showed much lower levels in the steady-state uninfected lymph nodes (as independently reported elsewhere^48^) and did not upregulate CD103 on infection in the same manner as BALB/c mice (Figure 4b). Similarly, fewer SJL Tregs upregulated GATA3 in response to infection (Figure 4c), which may be important as GATA3 has been found to be required for functional Treg suppression in the gut.^49^ These data argue that although there is no correlation between susceptibility and the overall numbers of Foxp3^+^ Tregs, a qualitative distinction may exist in the suppressive capacity of these cells in the most resistant SJL genotype.

We next examined lamina propria (LP) Treg populations, focussing on a comparison of SJL and C57BL/6 mice. Again, total frequencies of Foxp3^+^ Tregs did not differ significantly between the strains and showed little proportional change as a result of infection (Figure 4d). However, while CD103 expression levels among uninfected LP Tregs were broadly similar in the two strains, by 7 days following infection CD103 expression within the Foxp3^+^ compartment was more than twofold higher in the susceptible C57BL/6 mice than in the resistant SJL (Figure 4e).

As CD103 induction is associated with TGF-β stimulation, and the known importance of TGF-β in *de novo* conversion of peripheral T cells to a regulatory phenotype, we also compared the ability of SJL and C57BL/6 T cells to convert from Foxp3^–^ to Foxp3^+^ T cells on incubation with mammalian TGF-β, or with HES which contains a functional mimic of TGF-β.^22^ As shown in Figure 4f, both C57BL/6 and SJL T cells were able to upregulate Foxp3 expression, although the proportional increase was less marked in cells from the resistant SJL strain.

### Resistant mice mount faster and broader specific antibody responses

Specific IgG1 antibodies are known to be protective against *H. polygyrus* infection,^30, 31, 32, 33, 34^ and their titer correlates with genetic resistance to this parasite.^50^ We assayed serum antibody responses to parasite excretory–secretory products (termed HES) that are known to be the primary targets of serum antibodies in infected mice.^51^ Anti-HES IgG1 antibody titers were found to be 10-fold higher in the resistant SJL strain (Supplementary Figure 4A), with a broader repertoire of antigen recognition (Supplementary Figure 4B), compared with the other strains. Surprisingly, specific IgE production was significantly higher in the most susceptible strain, CBA (Supplementary Figure 4C) and relatively low, as previously noted,^52^ in resistant SJL mice. No difference in anti-HES IgA titers were observed between strains (data not shown). *H. polygyrus* infection has also been reported to stimulate hyper-IgG and -IgE serum levels reflecting polyclonal antibody stimulation.^30, 53, 54^ We noted that while total serum IgG1 levels rose >10-fold in infected mice of all strains (Supplementary Figure 4D), SJL mice showed almost no increment in serum IgE concentrations, unlike the other three strains (Supplementary Figure 4E). Overall, these data reinforce the conclusion that IgG1, rather than IgE, is the critical isotype required for immunity to *H. polygyrus*.^33^

### Resistance to primary infection is associated with alternative activation of macrophages

**Figure 5:**
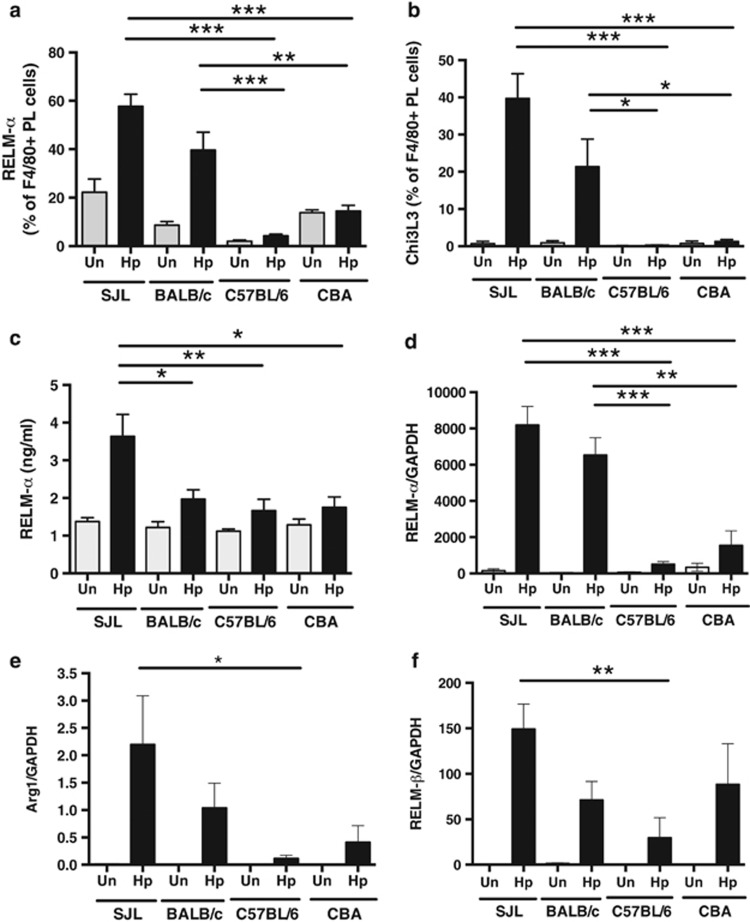
Expression of alternative activation genes in infected animals. All results are from two experiments pooled with 4–6 mice per group represented as mean values with standard error. (**a**, **b**) RELM-α and Chi3L3 (Ym1) expression by peritoneal macrophages of naive and d7 infected mice. (**c**) Expression of RELM-α protein in duodenal tissue homogenate of naïve and d7 infected mice by ELISA. (**d**–**f**) Quantification of RELM-α, Arginase-1 and RELMβ in duodenal tissue of naïve and d7-infected mice by real-time PCR, relative to the housekeeping gene GAPDH. Statistically significant differences are indicated; *P<0.05; **P<0.01; ***P<0.001.

Many helminth infections drive a specialized functional program in macrophages termed as alternative activation, associated with expression of distinct gene products including Arginase-1, Chitinase-3-like protein 3 (also known as Ym-1) and Resistin-like molecule-α (RELM-α, otherwise FIZZ-1).^55^ These markers of alternative activation were strikingly elevated among the expanded peritoneal macrophage populations in the SJL and BALB/c strains, in which 20–40% expressed Chi3L3 or RELMα within 7 days of infection (Figures 5a and b). These gene products were also upregulated in intestinal tissue, as found for RELM-α protein both by ELISA (Figure 5c) and RT-PCR (Figure 5d). Similarly, Arginase-1 mRNA levels were highest in the SJL strain (Figure 5e). Expression of RELM-β, which has been reported to exert a direct anti-parasite effect on *H. polygyrus*,^56^ was also maximal in infected SJL mice, although in this case all strains induced expression following infection (Figure 5f). Other myeloid phenotypes are also expanded in the peritoneal cavity of infected mice, including CD11b^+^Gr1^+^ cell populations (data not shown), again displaying a gradient matching the resistance status of the host.

### Granulomas in resistant mice have high levels of alternative activation

**Figure 6:**
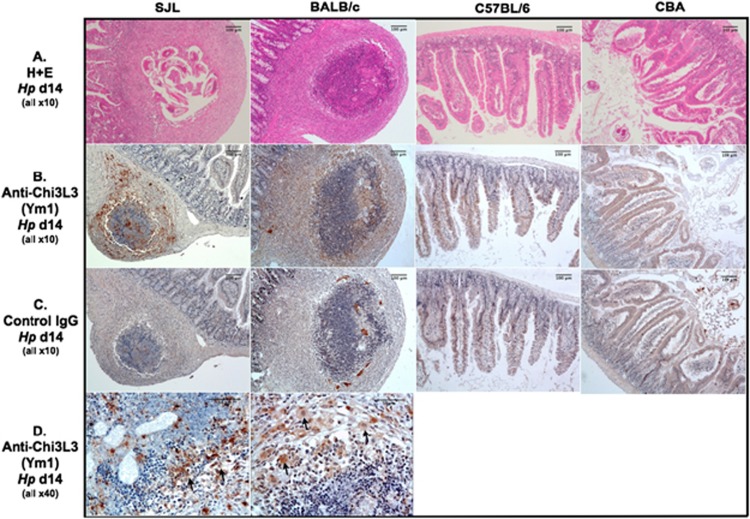
Primary resistance to *H. polygyrus* infection is associated with granuloma-like formation in the gut wall expressing Chi3L3. Histological sections of granulomas and normal intestinal tissue from *H. polygyrus*-infected mice. All images in (**a**–**c**) were captured and are presented at the same magnification. (Row **a**) Hematoxylin and eosin staining of sections from mice 14 days following infection; note presence of trapped larvae in granuloma from SJL mouse. Scale bars show 200 μm. (Rows **b**, **c**) Staining with anti-Chi3L3 and control antibodies in sections from the same mice. Scale bars show 200 μm. (Row **d**) Staining with anti-Chi3L3 in SJL and BALB/c mice; note both staining of diffuse protein within the granuloma and within large mononuclear cells (indicated by arrows). Scale bars show 200 μm. A full colour version of this figure is available at the *Immunology and Cell Biology* journal online.

A notable feature of *H. polygyrus* infection in the more resistant animals is the development of numerous macroscopic granulomas in the gut wall by day 14 (Figure 1e). Similar granuloma-like structures have been reported following secondary challenge of susceptible mice that have been cleared of primary infection by curative drug treatment,^11, 29, 57^ as well as in resistant mice within the course of primary infection itself^35^; these granulomas have been reported to be macrophage-rich with a significant influx of neutrophilic granulocytes.^58^ Histological analysis of the duodenal wall showed that at day 14 post infection, some granulomas in SJL mice still contained parasites although the great majority had emerged into the lumen (Figure 6a). At this time point, all parasites in the other strains were found in the lumen.

To assess the level of alternative activation *in situ*, sections of gut wall (containing granulomas in the appropriate strains) were probed with antibody to the Chi3L3 (Ym1) protein product. As also shown in Figure 6b, SJL mice showed high levels of Ym1 protein both in and around the granuloma, often associated with large mononuclear cells (Figure 6d arrows). No significant staining was observed in the two more resistant strains, or in gut tissues taken from uninfected mice of any genotype (data not shown).

### Clodronate depletion of macrophages inhibits intestinal granulomas but does not completely negate immunity

**Figure 7:**
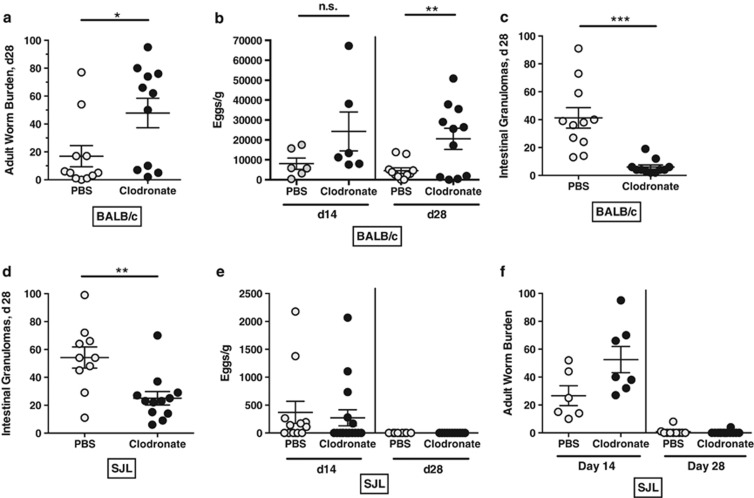
Clodronate depletion of macrophages in BALB/c and SJL mice infected with *H. polygyrus*. All results are pooled from two experiments and bars represent the mean and standard error. Data showing the efficacy of clodronate depletion are presented in Supplementary Figure 5. (**a**) Adult worm burdens from female BALB/c mice treated i.v. with either PBS or clodronate liposomes and infected for 28 days with *H. polygyrus.* (**b**) Egg burden in feces from female BALB/c mice treated i.v. with either PBS or clodronate liposomes, at day 14 and 28 after infection with *H. polygyrus.* (**c**) Intestinal granuloma counts from female BALB/c mice treated i.v. with either PBS or clodronate liposomes and infected for 28 days with *H. polygyrus*. (**d**) Intestinal granuloma counts from female SJL mice treated i.v. with either PBS or clodronate liposomes and infected for 28 days with *H. polygyrus.* (**e**) Egg burden in feces from female SJL mice treated i.v. with either PBS or clodronate liposomes, at day 14 and 28 after infection with *H. polygyrus.* (**f**) Adult worm burdens from female SJL mice treated i.v. with either PBS or clodronate liposomes and infected for 14 or 28 days with *H. polygyrus.* Statistically significant differences are indicated; *P<0.05; **P<0.01; ***P<0.001.

Clodronate liposome administration was then used to deplete phagocytic immune cells *in vivo* during the first week of *H. polygyrus* infection. In BALB/c mice, clodronate treatment, which showed >80% depletion of circulating CD115^+^ monocytes (Supplementary Figure 5), significantly impaired immunity as shown by increased adult worm (Figure 7a) and egg (Figure 7b) numbers at day 28. At this later time point, few granulomas were present in clodronate-treated mice (Figure 7c). Similarly, clodronate-treated SJL mice formed a reduced number of intestinal granulomas (Figure 7d), but continued to repress egg production (data not shown), while adult worm expulsion was only slightly delayed (Figure 7e). Hence, the strength of the contribution of clodronate-sensitive phagocytes to protection against helminth infection depends on the genetic background of the host.

## Discussion

Immunity to gastrointestinal helminth infections requires the appropriate and co-ordinate responsiveness of the innate and adaptive immune systems.^8, 11, 59^ The degree to which immunity successfully excludes the parasite varies, however, according to the genetic status of the host; thus, comparisons of genetically susceptible and resistant genotypes can identify key components and mediators that are required for most effective immune protection.

In this study, we have focussed on the helminth *H. polygyrus*, which is a natural parasite of the mouse, and to which different mouse strains show diverse patterns of susceptibility to infection,^36, 38^ with resistance clearly associating with strength of Th2 responsiveness.^40, 60, 61^ Our new data highlight the significance of early events in infection, as by day 14 post inoculation, dramatic differences were apparent in worm fecundity between the strains, which preceded subsequent worm expulsion in the resistant genotypes. Those strains able to limit egg production and curtail infection display a suite of enhanced Type 2 responses, including in particular T cell production of IL-4 and IL-10, eosinophilia and alternatively activated macrophages. Previous studies have highlighted such components in individual strains, for example showing rapid T-cell-independent type 2 cytokine responses in infected BALB/c mice^62^ but these have not previously been correlated to the differential susceptibility of inbred mouse strains.

The positive correlation between levels of the cardinal type 2 cytokines IL-4 and IL-13, and early expulsion of *H. polygyrus*, is entirely consistent with published reports on the strength of Th2 responses in resistant mice. More surprising however is that IL-10 not only parallels the major Th2 cytokines but shows a more extreme polarization: thus, the more resistant strains express the highest IL-10 when judged by elevated intracellular cytokine staining and by antigen-specific recall responses *in vitro*. Poor, or slow, IL-10 production could permit higher IFN-γ levels among both CD4^+^ and CD8^+^ T cells in the susceptible mice (and possibly IL-17 in the C57BL/6 mouse), which feed back to dampen the Th2 response more broadly. An interesting possibility is that IL-10 acts in mice in a manner similar to that in helminth-infected humans, promoting an IgG isotype (IgG1 in mice, IgG4 in humans) while suppressing IgE. In contrast to the clear association of IL-10 responsiveness with resistance, IL-17 expression did not correlate with any infection outcome in this study, suggesting that in primary infection at least, this compartment of the immune system is not a critical factor.

Perhaps the most striking feature of the BALB/c and SJL strains is the extensive number of macrophage-rich granulomas in the intestinal wall that (as shown in SJL mice) envelop the larval stage of the parasite. Such granulomas appear to be similar to those described in secondary infection, in which it was suggested that AAMs in the granulomas had a key role in mediating parasite killing^11, 12, 29^; the granulomas have also been reported to require IL-21.^63^ We established that the primary granulomas are similarly macrophage-dependent, and associated with alternative activation, by three independent approaches. First, clodronate depletion reduced granuloma formation; secondly, gene expression of AAM markers Arginase-1, Chi3L3 (Ym1) and RELMα was maximal in the resistant SJL strain. Finally, granulomas were absent in the IL-4Rα-deficient setting, in which alternative activation of macrophages does not occur in response to helminths.^64, 65^

A further factor in the resistance of SJL mice may be a subtle defect in the activation of regulatory T cells; this may not be evident solely in helminth infection, as the SJL mouse is also prone to a series of autoimmune conditions, and indeed defective T-cell suppression has long been noted in this strain.^66^ At this stage, our data are purely indicative of a correlative link between Treg activation and the response to infection, although such an association has been found in a recent study of *H. polygyrus* infection in IL-6-deficient BALB/c mice.^67^ If SJL mice are deficient in inducible Tregs, this may allow them to more rapidly deploy alternatively activated macrophages, as it has recently been reported that mice lacking the conserved nucleotide sequence CNS-1 in the Foxp3 gene (which allows TGF-β-mediated signals to stabilize Treg function), and lacking inducible Tregs, display uncontrolled accumulation of Chi3L3-expressing alternatively activated macrophages.^68^ Future studies should explore the role not only of Tregs in this setting, but regulatory B cells that were previously found to be active in *H. polygyrus* infections of the more susceptible C57BL/6 strain.^69^

An additional cell type that has been proposed to be important in immunity to *H. polygyrus* is the mast cell,^70^ albeit as a source of cytokines rather than through a direct killing mechanism. As IL-9 over-expression is associated with enhanced immunity to *H. polygyrus*,^10^ we measured levels of the key cytokine IL-9 between the strains, but found no difference. Possibly, as noted previously, mast cells in SJL mice are more active than in other genotypes,^71^ and this feature remains to be further investigated. Finally, our data show that innate lymphoid cells can expand and produce type 2 cytokines following infection, and are present in the MLN of more resistant genotypes. Previously, in studies of *H. polygyrus*-infected C57BL/6 mice, such cells were reported to be absent from the MLN but detectable in the LP^70^; in our own unpublished studies we found LP ILC2s in both infected SJL and C57BL/6 mice but at similar levels to those in naive controls, indicating that a key difference between the strains may be the expansion of these cells in the draining lymph node.

A key question now is whether protective immunity can be successfully mediated towards *H. polygyrus* after it has emerged from its tissue-dwelling phase. As demonstrated, resistant strains of mice were able to negatively affect worm fecundity early in infection. It is likely that once they have emerged into the lumen, adult worms are no longer susceptible to attack by macrophages and other myeloid cells. This is supported by studies in which secondary protection to *H. polygyrus* could be circumvented if adult worms were directly planted into the duodenum.^72^ However, the damaging effect of macrophages in the granuloma may be manifest first only as reduced fecundity, but eventually leads to death and/or expulsion of the adult worms some days later. This model would be consistent with multiple mechanisms of attack, including macrophage-independent effects as were evident in clodronate-treated SJL mice in our study and also in clodronate-treated BALB/c immune mice, which although unable to expel adult worms, still reduced fecundity in the parasite population.^29^ However, as the luminal adults are immunosuppressive, if they are not sufficiently degraded by the time of emergence, they may survive through their strong immuno-regulatory effects as seen in a number of chronic infection settings.^18, 73, 74^

In conclusion, the perspective we present here is one of broad type 2 activation being required, extending to macrophages and ILC2 cells as well as conventional CD4^+^ T lymphocytes, within a strongly quantitative setting in which the rapidity and peak level of the type 2 response are critically important for the establishment of immunity. However, the gradient of immune effects over genotypes and over time also reflects an incremental character to anti-helminth immunity that could usefully be targeted by interventions designed to boost protection in the infected host, an objective that would be enormously valuable to the treatment of helminth infections worldwide.

## Methods

### Mice

CBA, C57BL/6, BALB/c, and SJL mice, IL-4R^–/–^ BALB/c mice^75^ and IFNγ^–/–^ B6 mice were bred in-house and housed in individually ventilated cages according to UK Home Office guidelines. Infections employed 200 L3 larvae of *H. polygyrus bakeri* maintained as previously described.^51^

### Clodronate treatment and antibody depletion

Clodronate liposome treatment was conducted as described elsewhere,^29, 76^ administering 200 μl clodronate i.v. on days 0, 1, 3 and 6 of infection, with peripheral blood sampling at day 7. For CD8^+^ T-cell depletion, C57BL/6 mice were injected with 200 μg anti-CD8α clone YTS169 i.p. on days −1, 0, 2, 5, 7, 9, 12, 14, 16, 19, 21, 25 and 27, before experimental harvest at day 28. Control mice received rat IgG (Sigma, Gillingham, Dorset, UK).

### Lymphocyte recovery, *in vitro* culture

MLN cell suspensions were prepared directly by passage through 70 μm nylon filters (BD Biosciences, Oxford, UK) and placed in complete RPMI1640 medium (cRPMI) containing 10% FCS, 100 U ml^−1^ penicillin, 100 μg ml^−1^ streptomycin and 2 mM L-glutamine. Peritoneal exudate cells were collected by washing the peritoneal cavity with cRPMI using a 23-gauge needle.

For isolation of LP cells, following removal of Peyer's Patches, intestines were opened longitudinally, rinsed and transferred to ‘3% buffer' (RPMI with 100 U ml^−1^ penicillin, 100 μg ml^−1^ streptomycin, 20 mM HEPES and 2 mM EDTA). Intestines were sliced into ∼1 cm pieces, which were disrupted by shaking by hand for 30 s, after which the buffer was poured off and replaced with fresh media. This was carried out three times after which the gut pieces were incubated at 37 °C in 20 ml 3% buffer containing 10% FCS, 5 mM EDTA and 0.145 mg/ml DTT (Sigma). The shaking and washing steps were repeated after which the intestine was homogenized with scissors into 10 ml RPMI containing 100 U ml^−1^ penicillin, 100 μg ml^−1^ streptomycin, 20 mM HEPES, 1% NEAA, 1% sodium pyruvate, 2 mM L-glutamine, 0.1% 2-mercaptoethanol, 5 mg Liberase TL (Roche, Burgess Hill, West Sussex, UK) and 25 mg DNAse 1 (Sigma). After 25 min of incubation with stirring at 37 °C, cells were pushed through a 70-μm filter followed by a 40-μm filter to remove debris.

Cells were then either stained for flow cytometry or restimulated in cRPMI with 1 μg ml^−1^ *H. polygyrus* adult Excretory-Secretory (HES) antigen,^77^ anti-CD3 or medium alone for 72 h at 37 °C, and cytokine production measured by ELISA.

### Cytokine assays

Cytokine levels were detected in culture supernatants by ELISA using monoclonal capture and biotinylated detection antibody pairs as follows: for IL-4, 11B11 and BVD6-24G2; IL-10, JES5-2A5 and SXC-1; IL-9, D8402E8 and D9302C12; IL-13, eBio13A and eBio1316H; IL-17, TC11-18H10 and TC11-8H4.1; IFN-γ, R46A2 and XMG1.2. All were purchased from BD Biosciences or eBioscience (Hatfield, UK), except 11B11 and R46A2, which were produced in-house. Standard ELISA conditions were employed, and following development of plates with streptavidin-alkaline phosphatase and *p*-nitrophenyl phosphate substrate, cytokine concentrations were determined by reference to a standard curve of doubling dilutions of a reference standard.

### Flow cytometry

Flow cytometry analyses were performed with PBS containing 0.5% bovine serum albumin (BSA) (Sigma) and 0.05% sodium azide (Sigma). Cells were stained in 96-well round-bottomed plates. Prior to FACS antibody staining of cells, Fc receptors were blocked in 50 μl of FACS buffer containing 100 μg ml^−1^ of naive rat IgG (Sigma) for 20 min at 4 °C. Samples were then washed in 200 μl of FACS buffer and surface stained for 20 min in 20 μl of FACS buffer containing a combination of the following antibodies: CD4 (RM4-5 or GK1.5; Biolegend, Cambridge, UK), CD8 (53-6.7; Biolegend), CD11b (M1/70; Biolegend), F4/80 (BM8; Biolegend), SiglecF (E50-2440; BD), CD115 (AFS98; eBioscience), CD103 (M290; BD), ICOS (15F9; eBioscience), CD3 (17A2; Biolegend), CD5 (53-7.3; Biolegend), CD11c (N418; Biolegend), CD19 (6D5; Biolegend), CD127 (A7R34, Biolegend), T1/ST2 (DJ8; MD Bioproducts) and Ly6C (HK1.4; Biolegend). To measure intracellular cytokines, cells were first stimulated for 4 h at 37 °C in the presence of PMA (50 ng ml^−1^) (Sigma), Ionomycin (1 μg ml^−1^) (Sigma) and Brefeldin A (10 μg ml^−1^) (Sigma). Following surface staining, cells were permeabilized for 30 min at 4 °C in Cytofix/Cytoperm solution (BD), and then washed twice in 200 μl of Perm/Wash (BD). Cells were stained for intracellular cytokine expression in the same manner as for surface markers but substituting perm/wash for FACS buffer. Intracellular cytokine stains used were IL-4, 1B11 (Biolegend); IL-10, JES5-16E3 (BD); IL-17, TC11-18H10 (BD); IFN-γ, XM6-1.2 (Biolegend); IL-13, eBio13A (eBiosciences) or appropriate isotype controls.

For Foxp3, GATA-3, RELM-α and Ym-1 samples were stained for surface markers after which cells were permeabilized for up to 12 h at 4 °C in Fix/Perm solution (eBioscience Foxp3 staining set), and then washed twice in 200 μl of Perm/Wash (eBioscience Foxp3 staining set). Cells were stained for Foxp3 (FJK-16 s; eBioscience), GATA-3 (L50-823; BD), RELM-α (226033; R&D, and rabbit IgG AF647 labeling reagent kit; Invitrogen) and Ym1 (biotinylated goat anti-mouse Chitinase 3-like 3; R&D, and Streptavidin-PeCy7; Biolegend) expression in the same manner as for surface markers but substituting Perm/Wash for FACS buffer.

After staining cells were washed twice in 200 μl of FACS buffer before acquisition on the LSR II or Canto flow cytometer (BD Bioscience), and subsequently analyzed using FlowJo (Tree Star, Ashland, OR, USA).

### Histology and immunostaining

Transverse sections were made from 2 cm of paraffin-embedded small intestine, at a thickness of 4 μm using a microtome. Sections were directly stained with hemotoxylin and eosin or processed for immunostaining with anti-Ym1 antibodies. Briefly, sections were deparaffinized by immersing slides in Histoclear (Brunel Microscopes Ltd) for 5 min, and then hydrated through 100%, 95% and 70% ethanol successively. Antigen retrieval was carried out by immersing slides in citrate buffer (20 mM citric acid, 0.05% Tween 20 at pH 6.0) warmed to 95 °C for 20 min. Slides were washed twice in 1 × PBS, sections ringed with a wax pen and 200 μl block (1 × PBS+1% BSA, 2% normal rabbit serum, 0.1% Triton X-100 and 0.05% Tween 20) added for 30 min at room temperature.

Rat α-mouse Ym1 (R&D Systems, Abingdon, UK) or rat IgG control (Sigma) was added at 25 μg ml^−1^ in block buffer and left overnight at 4 °C. Slides were immersed in 3% H_2_O_2_ for 10 min at room temperature, and washed in PBS. Rabbit α-rat IgG conjugated to biotin (Vector Laboratories, Peterborough, UK) at 5 μg ml^−1^ in PBS was added for 1 h at room temperature, in the dark. Following 2 washes in PBS, several drops of ABC Vectastain (Vector Laboratories) were added and slides left for 30 min at room temperature, in the dark. Slides were washed twice in PBS and DAB peroxidase solution (Vector Laboratories) was added for 5 min (until a brown stain had developed).

With water washes in between, the following were added successively to counterstain the sections: Harris hemotoxylin solution (Sigma), acid alcohol (75% ethanol, 1% HCl) and Scott's Tap Water Substitute (ddH_2_O+42 mM NaHCO_3_ and 167 mM MgSO_4_). Slides were dehydrated through 75, 95 and 100% ethanol and then Histoclear added for 5 min. Coverslips were added with DPX mountant (Sigma) and slides were left to dry overnight, in the dark. Pictures were taken using a Leica DFC290 compound microscope and Leica Application Suite software (Leica Microsystems, Milton Keynes, UK).

### Gut homogenate and ELISAs

Approximately 1 cm section of small intestine was homogenized in 500 μl 1 × lysis buffer (Cell Signaling Technology Inc, Hitchin, UK) plus 5 μl PMSF (Sigma) using a TissueLyser (Qiagen, Manchester, UK). Samples were centrifuged at 12 000 *g* for 10 min to remove debris and supernatants added to ELISAs to measure RELM-α content, using the antibody pair clone 226033 and biotinylated goat anti-mouse RELM-α (both R&D Systems).

### RT-PCR

Approximately 0.5 cm of the uppermost part of the duodenum was placed into 1 ml of TRIzol (Invitrogen, Paisley, UK) and extracted according to the manufacturer's protocol; 15 μl RNA was treated with DNAse (DNAFree kit, Ambion, Paisley, UK), concentrations were determined using a Nanodrop 1000 (Thermo Scientific, Hemel Hempstead, UK) and samples reverse-transcribed using 1–2 μg of RNA with M-MLV reverse transcriptase (Promega). A PCR block (Peltier Thermal Cycler, MJ Research) was used for the transcription reaction at 37 °C for 60 min. Gene transcript levels were measured by real-time PCR on a Roche Lightcycler 480 II, in 10 μl total volume made up of 4 μl cDNA, 5 μl SYBR Green (Roche), 0.3 μl of each primer (10 μM) and 0.4 μl DEPC treated water (Ambion) using standard conditions for 60 cycles. Target gene expression levels were normalized against the housekeeping gene GAPDH.

The forward and reverse primers used, and resultant amplicons, were as follows: Arginase-1, CAGAAGAATGGAAGAGTCAG and CAGATATGCAGGGAGTCACC (249 bp); GAPDH, ATGACATCAAGAAGGTGGTG and CATACCAGGAAATGAGCTTG (112 bp); RELM-α, TATGAACAGATGGGCCTCCT and GGCAGTTGCAAGTATCTCCAC (107 bp); RELM-β, GGAAGCTCTCAGTCGTCAAGA and GCACATCCAGTGACAACCAT (105 bp).

### Antibodies

Whole blood was clotted overnight at 4 °C and the fluid phase centrifuged to remove remaining RBC. Serum was subsequently added in serial dilutions to ELISA plates coated with either 1 μg ml^−1^ HES, goat α-mouse Ig (Southern Biotech, Cambridge, UK) at 1 μg ml^−1^ or anti-IgE (clone R35-72, BD Biosciences) at 1.5 μg ml^−1^ in carbonate buffer. Antibody binding was detected using HRP-conjugated goat anti-mouse IgG1 or IgE (both Southern Biotech) and ABTS Peroxidase Substrate (KPL, Wembley, UK), and read at 405 nm. Immunoprecipitations with biotin-labeled HES were performed with sera as described previously.^51^

### Statistical analysis

All statistical analyses were performed using Prism 5 (Graphpad Software Inc, La Jolla, CA, USA). For comparisons of two groups Student's *t*-test was used. When three or more groups were analyzed then a one-way ANOVA was used with a Tukey's multiple comparison test. *P*-values of <0.05 were considered to be significant.
