# Supplementary material for: Innate and adaptive type 2 immune cell responses in genetically controlled resistance to intestinal helminth infection
Source: Immunol Cell Biol. 2014 Feb 4;92(5):436–48. doi: 10.1038/icb.2013.109 (PMC4038150; doi:10.1038/icb.2013.109)

Supplementary Figures

Supplementary Figure 1

Intracellular cytokine profile of MLN T cells between 7 and 28 days of *H. polygyrus* infection of different mouse strains.

MLNC from day 7, 14 or 28 *H. polygyrus*-infected female SJL, BALB/c, C57BL/6 and CBA mice were stained after 4 hours of stimulation with Brefeldin, PMA and ionomycin for intracellular cytokines. Data represent mean and standard error values from 4 (naïve) or 6 (infected) individual mice of each strain, and are pooled from 2 independent experiments.

A-E. IL-4, IL-10, IL-13, IL-17 and IFN-γ staining of MLN $CD4^+$ T cells

F. IFN-γ staining of $CD8^+$ T cells

Supplementary Figure 2

Antigen-specific cytokine response profile of T cells between 7 and 28 days of *H. polygyrus* infection of different mouse strains.

MLNC from day 7, 14 or 28 *H. polygyrus*-infected female SJL, BALB/c, C57BL/6 and CBA mice were stimulated in vitro with medium alone (Med) or 1 µg/ml HES, and supernatants assayed for secreted cytokines. Data represent mean and standard error values from 4 (naïve) or 6 (infected) individual mice of each strain, and pooled from 2 independent experiments. Negative control cultures of MLNC from uninfected mice stimulated with HES did not produce signficant levels of any cytokine.

A-E. IL-4, IL-10, IL-13, IL-17 and IFN-γ from day 7, 14 and 28 MLNC

F. IL-9 from d 14 MLNC

Supplementary Figure 3

Profile of mesenteric lymph node innate lymphoid cells

Single cell suspensions of mesenteric lymph nodes from naïve and 10-day *H. polygyrus* (Hp) infected BALB/c and C57BL/6 mice were stained with anti-CD127, ICOS, T1/ST2 and GATA3, together with a panel of lineage markers (CD3, CD5, CD11c, CD19).

A. Identification of Lin^-CD127^+ MLNC

B. GATA3 expression in Lin^-CD127^+ MLNC

C. T1/ST2 and ICOS expression in $GATA3^+$ MLNC

Supplementary Figure 4

Humoral responses to *H. polygyrus* infection.

Serum antibody responses were measured to HES (**A, C**), specific targets of antibodies were assayed by immunoprecipitation of HES (**B**) and total immunoglobulin G1 and E levels determined (**D, E**).

A. Anti-HES IgG1, measured by ELISA with *H. polygyrus* adult excretory-secretory antigen. Endpoint titers were determined for day 28 sera from individual mice and mean values ± standard errors are presented; data are representative of several replicate experiments.

B. Specificity of serum antibodies of naive and 28-day infected mice profiled by immunoprecipitation of biotinylated HES followed by binding of enzyme-linked streptavidin.

C. Anti-HES IgE, as for panel A.

D-E. Total serum IgG1 and IgE levels in naïve and 28-day infected mice of each strain; mean values ± standard errors are presented for sera from individual mice.

Supplementary Figure 5

Depletion of myeloid cells with clodronate

Peripheral blood was samples 7 days following administration of 200 µl of clodronate liposomes or PBS, injected i.v. at days 0, 1, 3 and 6 of *H. polygyrus* infection. Depletion of monocytes/macrophages by clodronate treatment was assessed by levels of CD11b, CD115 and Ly6C cell staining. Data shown are from BALB/c mice, similar depletion was noted at the same time point with SJL mice.

A. Percentage of CD11b^{+}Ly6C^{+} peripheral blood monocyte cells

B. CD115 MFI among CD11b^{+}Ly6C^{+} blood cells.

Filbey *et al.*, Supplementary Figure 1

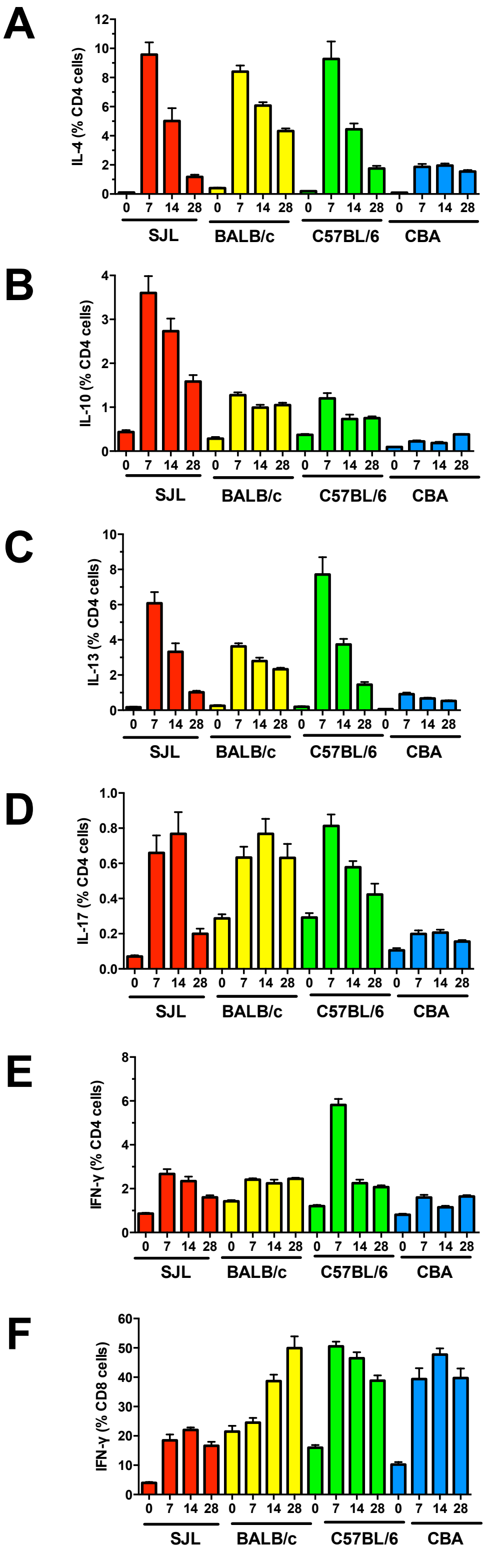

Filbey *et al.*, Supplementary Figure 2

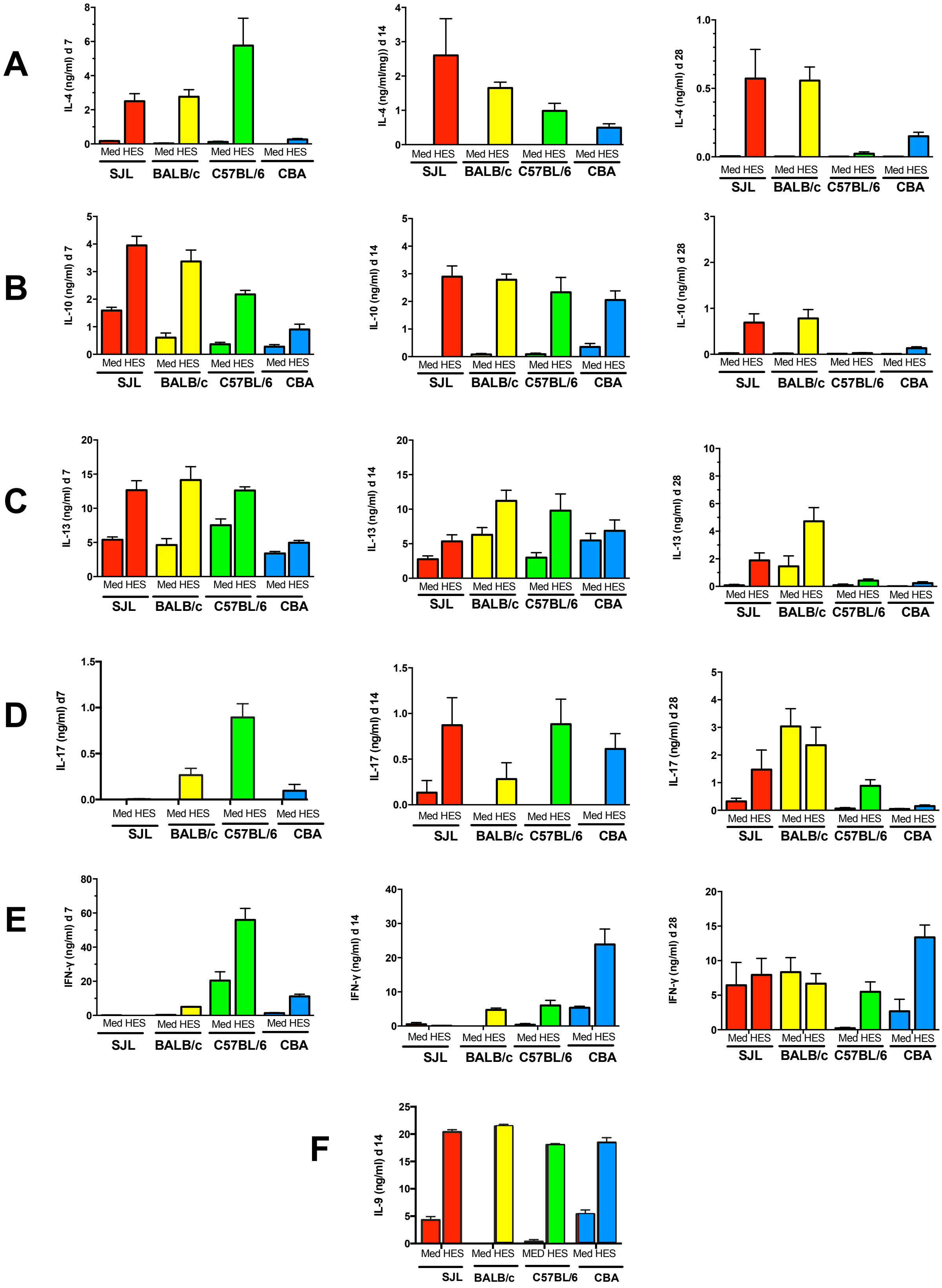

Filbey *et al.*, Supplementary Figure 3

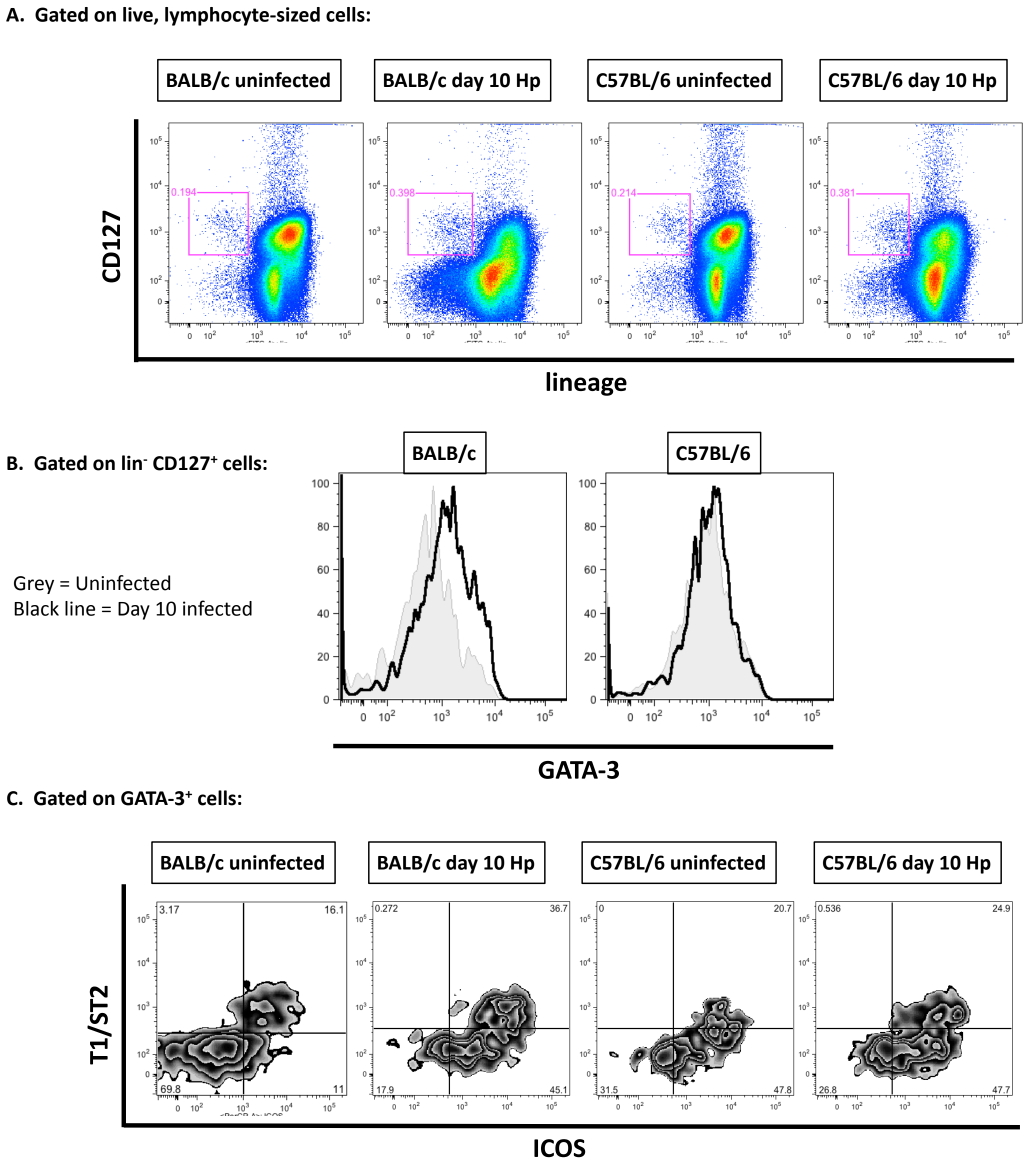

Filbey *et al*, Supplementary Figure 4

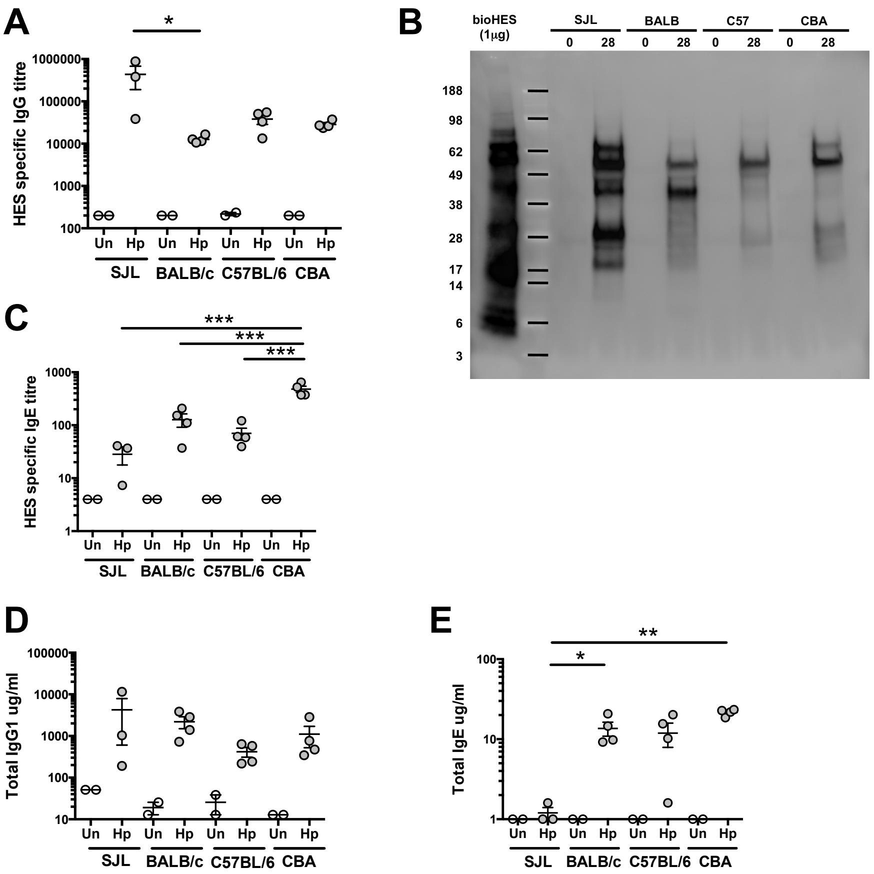

Filbey et al, Supplementary Figure 5

BALB/c

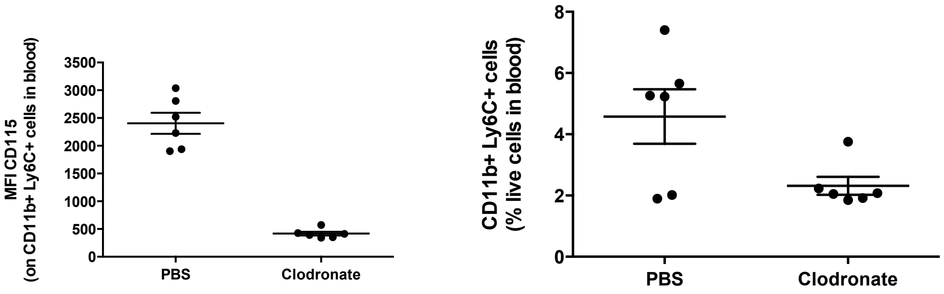

SJL

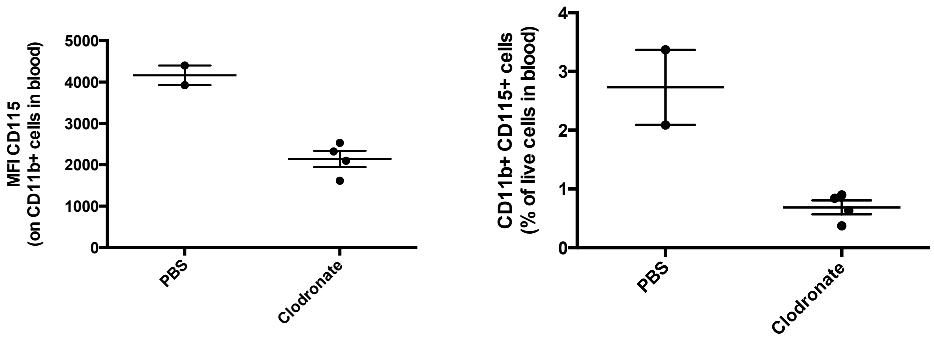

Supplement: Supplementary Figures [file icb2013109x1.pdf]
